# Effect of teachers’ teaching strategies on students’ learning engagement: moderated mediation model

**DOI:** 10.3389/fpsyg.2024.1475048

**Published:** 2024-12-03

**Authors:** Heping Zhang, Junjie Yang, Zhiyuan Liu

**Affiliations:** ^1^Normal School of Vocational Techniques, Hubei University of Technology, Wuhan, China; ^2^School of English Education, Guangdong University of Foreign Studies, Guangzhou, China

**Keywords:** teachers’ teaching strategies, teachers’ emotional engagement, students’ learning engagement, structural equation modeling, mediating effect

## Abstract

**Introduction:**

This study explores the nuanced relationship between teachers’ teaching strategies and students’ learning engagement within online environments, considering the mediation by students’ perceptions of teachers’ emotional engagement and the moderation by teachers’ expectations.

**Methods:**

Employing a stratified sampling technique, data were collected from 1,200 Chinese primary and secondary students through the “Survey on Online Learning Engagement.” Structural equation modeling was applied to analyze the relationships among teaching strategies, emotional engagement, teachers’ expectations, and learning engagement.

**Results:**

The study found that teachers’ teaching strategies not only directly affect students’ learning engagement (*r* = 0.377^***^, *p* < 0.001), but also indirectly affect students’ learning engagement through students’ perception of teachers’ emotional engagement. It showed a significant mediating effect (indirect effect = 0.231^***^, *p* < 0.001). Additionally, teachers’ expectations exhibit a notable moderating effect on the relationship between perceived emotional engagement and learning engagement (*β* = 0.073^***^, *p* < 0.001). In other words, in addition to teachers’ teaching strategies, teachers’ emotional involvement and students’ perception of teachers’ emotional involvement are important factors affecting students’ learning involvement. This means that in online classroom teaching, the emotional transmission and experience between teachers and students play a role in the connection.

**Discussion:**

The study highlights the critical role of emotional connection in educational strategies, suggesting that teachers should focus on fostering emotional engagement alongside academic content. Balanced teacher expectations are recommended to facilitate a supportive learning environment that encourages higher levels of student engagement. Educators are advised to integrate emotional warmth and responsiveness into their teaching methods and to maintain realistic expectations to promote optimal learning outcomes.

## Introduction

Globally, the surge of educational reform is increasingly concentrated on augmenting student academic outcomes with unprecedented vigor (OECD, 2019a). Research and policy initiatives in leading educational systems, including those of China, the United States, and OECD member states, underscore a heightened prioritization of student engagement and academic achievement. China’s “Education Modernization 2035” policy emphasizes the “optimization of educational resource allocation, the stimulation of student interest and potential, and the promotion of proactive learning and deep engagement” (The State Council of the People’s Republic of China, 2019). In the United States, the “Every Student Succeeds Act” (U.S. Department of Education, 2015) advances personalized learning, professional development for educators, and data-driven decision-making to elevate student performance and comprehensive development (Dewaele and Li, 2021). Similarly, the European Union’s “European Education Area 2025 Action Plan” advocates for innovative teaching approaches and the cultivation of critical competencies to achieve high-quality, inclusive education (European Commission, Directorate-General for Education, Youth, Sport and Culture, 2021). These policy shifts converge on a globally pertinent issue: how to strategically deploy and scientifically refine essential educational elements—such as teaching strategies, teacher engagement, and learning methodologies—to foster active student participation, enhance learning efficiency, and secure academic success.

Students’ learning engagement, defined as a critical indicator of the time, effort, and psychological resources students invest in learning activities, has long been a focal point in educational research (Johar et al., 2023). Scholars have dissected the construct of engagement, identifying three dimensions: behavioral, emotional, and cognitive engagement (Fredricks and McColskey, 2012). Kirschner et al. (2006) highlighted the essential role of teaching strategies, such as direct instruction, in enhancing students’ learning engagement. Additionally, Benson (2012) and Fredricks et al. (2004) emphasized that engagement is intricately linked to external factors, including sociocultural influences and the school environment. Gharavi et al. (2013), along with Ryan and Deci (2000), the founders of Self-Determination Theory (SDT), underscored the profound impact of learners’ psychological well-being and psychological needs on engagement. It is evident that students’ learning engagement is influenced not only by internal individual factors, such as cognitive styles, self-efficacy, and goal setting, but also by external environmental factors, including teaching models, classroom climate, and sociocultural contexts.

Research has shown that explicit teaching strategies and teacher behaviors are crucial factors influencing students’ learning outcomes, academic achievement, and holistic development (Koçak et al., 2021). Strategies such as setting clear teaching goals, providing effective feedback, implementing differentiated instruction, and creating opportunities for deep learning have been found to significantly enhance student outcomes (Rosenshine, 2012; Wisniewski et al., 2020). In addition to these cognitive and teaching factors, researchers have also focused on the attitudinal and emotional dimensions of learning. Pekrun (2006) proposed the Academic Emotions Theory, highlighting the critical regulatory role of student emotions in the learning process. Dewaele and Li (2021) further revealed the importance of emotional engagement in overall student engagement, noting a correlation between students’ perceived teacher enthusiasm and their own engagement levels.

In summary, existing research has extensively confirmed the close relationship between teachers’ teaching strategies and behaviors and student engagement. However, there is a lack of exploration into the underlying mechanisms of how these teaching strategies and behaviors influence student engagement. This study aims to construct a theoretical model of these internal mechanisms from the perspective of social cognitive theory. Using data from large-scale surveys and employing structural equation modeling, we test the proposed theoretical model. On one hand, we analyze the mediating role of perceived teacher emotional engagement between teachers’ teaching strategies and student engagement. This aims to explain how students perceive positive emotions from teachers’ teaching behaviors and subsequently translate these perceptions into their own engagement. On the other hand, we explore how teacher expectations moderate this process. This study will provide a theoretical foundation for optimizing teachers’ classroom practices to enhance students’ learning outcomes.

### Literature review

Bandura’s (1991) social cognitive theory posits that environment, individual, and behavior interact with each other reciprocally. In other words, environmental factors, individual factors, and individual behaviors mutually influence one another. In the context of teaching, teaching strategies constitute a specific environmental factor. The strategies employed by teachers can influence students’ perceptions of teacher emotional engagement (e.g., viewing the teacher as dedicated, attentive and responsible). Clearly, these perceptions fall under the category of individual factors, while students’ learning engagement can be regarded as a behavioral factor in the learning context. According to social cognitive theory, we propose a theoretical hypothesis: teachers’ teaching strategies may influence students’ learning engagement by affecting students’ perceptions of teachers’ emotional engagement.

Some empirical studies have provided support for the aforementioned theoretical hypothesis. For instance, research has found that the external teaching environment and students’ perceptions and recognition of teachers’ emotional engagement, which constitute the learners’ internal psychological environment, are crucial factors influencing student engagement (Schunk and DiBenedetto, 2020). On one hand, high-quality and diverse online teaching strategies, such as the provision of various resources, the creation of authentic contexts, thought-provoking discussions, inspirational guidance from teachers, and timely evaluative feedback, can effectively stimulate students’ enthusiasm for learning and actively engage them in the learning process (Yu, 2022). These strategies can also fully activate and enhance students’ critical thinking and investigative enthusiasm, thereby further increasing their level of engagement (Bu et al., 2022). On the other hand, individual experiences in learning are significant psychological factors affecting student engagement. Positive emotions experienced by students can promote their engagement in learning (Lin et al., 2020).

**Figure 1:**
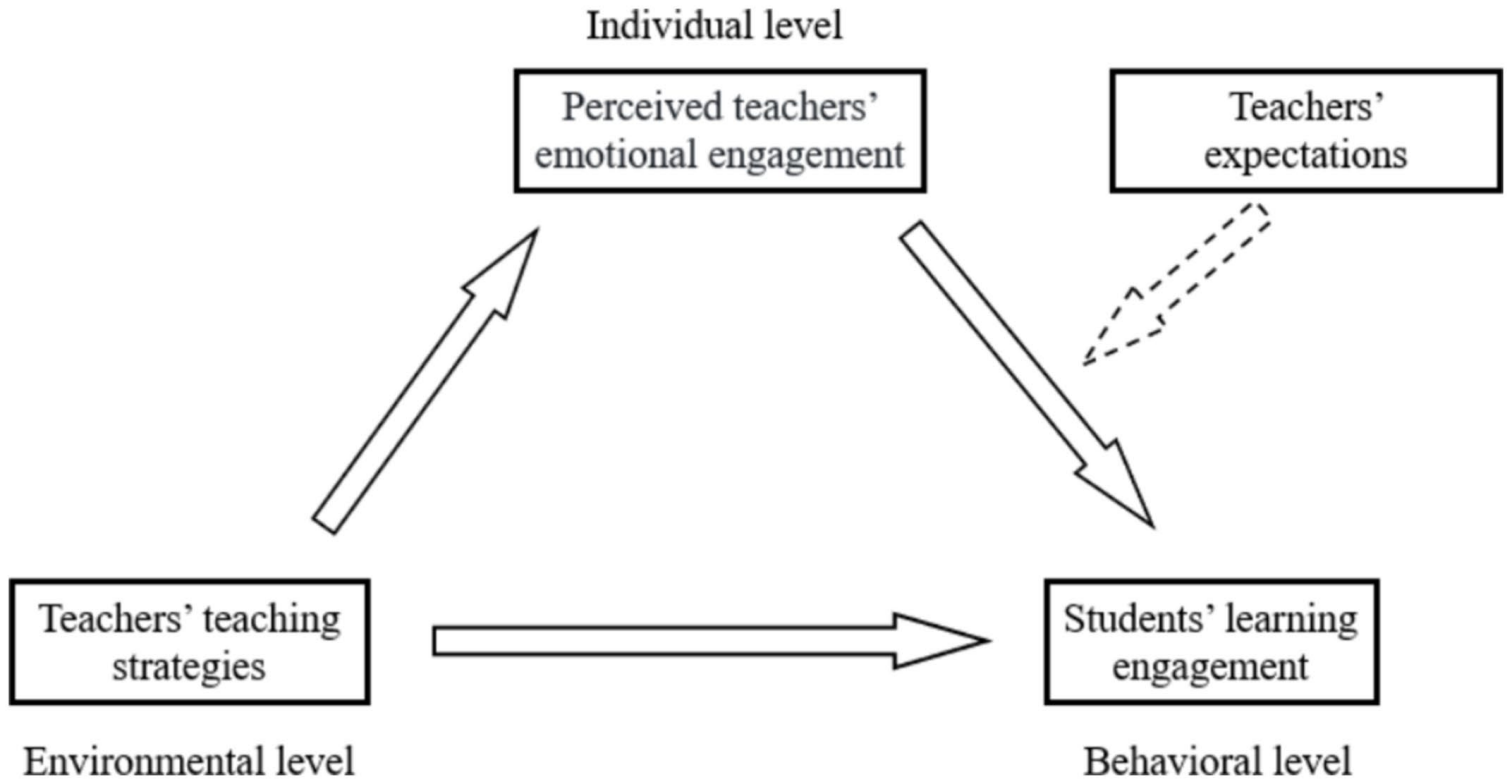
Conceptual framework.

In recent years, some empirical studies have focused on the mediating and moderating variables between teachers’ expectations and students’ learning engagement. For instance, Košir and Tement (2014) found that teachers’ expectations indirectly influence students’ learning engagement through students’ self-efficacy. Additionally, students’ perceptions of teachers’ expectations might moderate the relationship between teachers’ expectations and engagement (Gentrup et al., 2020). In our proposed theoretical hypothesis, teachers’ emotional engagement might positively impact students’ learning engagement, while students’ perceptions of teachers’ expectations could either strengthen or weaken this positive effect. Therefore, in our hypothesis model, we have included an examination of the moderating effect of teachers’ expectations on the relationship between perceived teachers’ emotional engagement and students’ learning engagement (see Figure 1).

In this model, teachers’ teaching strategies may directly influence students’ learning engagement or indirectly influence it through the mediating variable of students’ perceptions of teachers’ emotional engagement. Additionally, teachers’ expectations might moderate the relationship between perceived teachers’ emotional engagement and students’ learning engagement. Therefore, this study proposes the following three hypotheses:

Hypothesis 1: Teachers’ teaching strategies have a significant positive impact on students’ learning engagement.

Hypothesis 2: Perceived teachers’ emotional engagement mediates the relationship between teachers’ teaching strategies and students’ learning engagement.

Hypothesis 3: Teachers’ expectations moderate the relationship between perceived teachers’ emotional engagement and students’ learning engagement.

## Materials and methods

### Participants

The data for this study comes from the “Adolescent Online Learning Status Survey,” jointly designed and implemented by the Teacher Education Research Center and the Student Development Collaborative Research Center in Hubei Province, China. To mitigate the impact of COVID-19 on students’ studies, the Education Department of Hubei Province In January 2020, the Provincial Department of Education put forward the requirement of “stopping returning to school but not suspending classes” in the *Guiding Opinions on Carrying Out Online Teaching in Primary and Secondary Schools* during the epidemic prevention and control period. The survey began in May 2020, after the implementation of the requirement, at which point students in Hubei Province have experienced about 3 months of full online learning, and students have enough online learning experience. This survey targeted students across four stages: university (first to fourth year), high school (first to third year), middle school (first to third year), and elementary school (third to sixth grade). The online learning questionnaire covered topics such as family environment, personal characteristics, students’ learning behaviors, and teachers’ teaching behaviors during the pandemic. A total of 10,028 valid questionnaires were collected. This study focuses on analyzing data from primary and secondary school students, specifically elementary, middle, and high school stages, excluding university samples. This left 8,974 valid questionnaires, with 4,532 males (50.5%) and 4,442 females (49.5%). The distribution by educational stage is as follows: 4,854 elementary school students (54.1%), 2,826 middle school students (31.5%), and 1,294 high school students (14.4%).

### Measures

Students’ learning engagement is the dependent variable. The learning Engagement Scale in this study refers to the Work of Li and Huang (2010), who adapted to the learning characteristics of Chinese college students and made a localized revision based on the Utrecht Work Engagement Scale-Student (Schaufeli et al., 2002). They first translated the English scale and conducted a preliminary test, finding that the original scale had applicability problems in the Chinese cultural context. Then, the scale structure was optimized by adding items related to motivation, energy and concentration, adjusting the scoring method, and conducting a second test on 100 college students. The revised learning scale in this study consists of 5 items, including: “I am always energetic when studying online,” “I get distracted after a short while when studying online,” “I cannot resist the temptation of irrelevant online content when studying online,” “I need someone to urge me to complete my study tasks when studying online,” “It is relatively easy for me to persist in completing difficult study content.” These items measure students’ energy, focus, self-control, self-discipline, and perseverance. Responses were recorded using a four-point Likert scale: “strongly disagree,” “disagree,” “agree,” and “strongly agree,” reflecting different levels of engagement. The internal consistency of the scale was confirmed by a Cronbach’s alpha coefficient of 0.795. The Kaiser-Meyer-Olkin (KMO) measure of sampling adequacy was 0.783, and Bartlett’s test of sphericity was significant (*p* < 0.001), indicating high reliability and validity (Hopkins, 1998).

Teachers’ teaching strategies, the key independent variable, were measured using a scale adapted from the “Programme for International Student Assessment (PISA 2018)” focusing on teaching strategies. This scale encompasses seven dimensions: Goal Setting, Inspirational Guidance, Oral Assessment, In-depth Teaching, Tutoring and Q&A, Assignment of Homework, Tests and Exams. Responses ranged from “strongly disagree” to “strongly agree,” represented by numerical values from 1 to 4, indicating the extent to which these teaching strategies were employed. The scores for each item were averaged to reflect the overall comprehensiveness of teaching strategy usage, with higher scores indicating more comprehensive use. The internal consistency of this scale was confirmed by a Cronbach’s alpha coefficient of 0.792, indicating good internal consistency. The Kaiser-Meyer-Olkin (KMO) measure was 0.833, and Bartlett’s test of sphericity was significant (*p* < 0.001), showing high reliability and validity (Kim, 2013).

Students’ perceptions of teacher emotional engagement was the mediating variable. OECD PISA 2018 student questionnaire measures teachers’ interest and emotion in teaching. According to the definition of teachers’ emotional engagement in this paper, we refer to this measurement tool of PISA 2018 and choose two of the most relevant items to measure teachers’ emotional engagement (OECD, 2019b). The two items are: “I can feel that the teacher enjoys teaching us” and “The teacher’s enthusiasm motivates me to study seriously.” The responses ranged from “strongly disagree” to “strongly agree,” represented by numerical values from 1 to 4, indicating the degree of students’ perceived teachers’ emotional engagement. Since the scores of these items can be considered equally important, no additional weight adjustment was necessary. Therefore, in the data analysis, the sample values of these two items were summed, with higher total scores indicating stronger positive perceptions of teachers’ emotional engagement. The internal consistency of this scale was confirmed by a Cronbach’s alpha coefficient of 0.858, indicating high internal consistency.

Teachers’ expectations were measured using the item “The teachers at my school are very concerned about my learning.” Responses ranged from “strongly disagree” to “strongly agree,” represented by numerical values from 1 to 4, indicating the degree of students’ perceptions of teacher’s’ expectations.

### Statistical analysis

Based on the research data and hypotheses, the following analyses were conducted using IBM SPSS 27.0: standardization processing, normality test, common method bias test, descriptive analysis, scale reliability and validity tests, and variable correlation analysis. A structural equation model was established using IBM SPSS Amos 26.0 to analyze the mediating effect (Anderson and Gerbing, 1988; Blunch, 2012). The moderated mediation model, which includes the independent variable (teachers’ teaching strategies), the mediating variable (perceived teacher emotional engagement), the dependent variable (student engagement), and the moderating variable (teacher expectations), was tested using PROCESS v4.1.

## Results

### Common method bias test

To address potential common method bias, several control strategies were employed during the survey process. These strategies included conducting anonymous surveys and incorporating reverse-scored items. Besides, Harman’s single-factor test was commonly used to assess the presence of common method bias. The analysis identified 18 factors with eigenvalues greater than 1, with the first factor accounting for 34.73% of the total variance, which is below the critical threshold of 40%. This indicates that common method bias is not present in this dataset.

### Descriptive analysis

The results of the multivariate normality test indicated that the absolute values of skewness for the variables ranged from 0.03 to 0.98, and the absolute values of kurtosis ranged from 0.10 to 2.01, suggesting that the data approximated a normal distribution (Kim and Hodges, 2012). Descriptive analysis, as presented in Table 1, revealed several key insights: in the context of online learning, the mean scores for student self-discipline (2.90) and perseverance (2.84) were relatively low; among teaching strategies, the mean scores for assignment completion (2.59) and testing (2.38) suggested these strategies were employed relatively infrequently in online teaching; additionally, students rated teachers’ emotional engagement (6.48) and teachers’ expectations (3.19) highly.

**Table 1:** Descriptions of dependent and independent variables.

Variable/indicator	Mean	Standard deviation	Minimum	Maximum
Students’ learning engagement	14.56	3.21	5	20
Energy level	3.01	0.75	1	4
Focus attention	2.82	0.87	1	4
Self-discipline	3.00	0.95	1	4
Self-awareness	2.90	0.96	1	4
Willpower strength	2.84	0.78	1	4
Teachers’ teaching strategies	21.23	3.33	7	28
Goal setting	3.32	0.67	1	4
Inspiration guidance	3.35	0.66	1	4
Oral evaluation	3.37	0.65	1	4
Deep teaching	3.27	0.70	1	4
Q & A	2.94	0.81	1	4
Homework assignment	2.59	0.71	1	4
Quiz testing	2.38	0.78	1	4
Perceived teachers’ emotional engagement	6.48	1.35	2	8
Teacher enthusiasm	3.22	0.73	1	4
Teacher motivation	3.26	0.71	1	4
Teachers’ expectations	3.19	0.73	1	4

### Correlation analysis

Table 2 presents the correlation relationships among the four variables in this study. There is a significant positive correlation between teachers’ teaching strategies and students’ learning engagement (*p* < 0.001). Perceived teachers’ emotional engagement shows a significant positive association with both teachers teaching strategies and students’ learning engagement (*p* < 0.001). Additionally, teachers’ expectations have a significant positive correlation with students’ learning engagement, teachers’ teaching strategies, and perceived teachers’ emotional engagement (*p* < 0.001). Based on this analysis, there are indeed significant correlations among the research variables in the theoretical model. This finding supports the further exploration of the moderated mediation effect of teachers’ teaching strategies on students’ learning engagement.

**Table 2:** Correlation coefficients matrix of variables.

Variable/Indicator	1	2	3	4
1. Students’ learning engagement	–			
2. Teachers’ teaching strategies	0.377^***^	–		
3. Perceived teachers’ emotional engagement	0.425^***^	0.664^***^	–	
4. Teachers’ expectations	0.353^***^	0.571^***^	0.716^***^	–

^***^p < 0.001.

### Mediation effect analysis

To test Hypotheses 1 and 2, this study constructed a structural equation model (SEM) in IBM SPSS Amos 26.0 software based on the theoretical framework. The SEM examined both direct and mediating effects, taking into account potential residual correlations among variables. The baseline model assessed the impact of teachers’ teaching strategies on students’ learning engagement, while the mediating model incorporated the influence of perceived teachers’ emotional engagement on top of the baseline model. By comparing these two models, it was found that the influence of teachers’ teaching strategies on students’ learning engagement was significant, and there was evidence supporting the mediating effect of perceived teachers’ emotional engagement.

Based on Table 3, several indicators robustly demonstrate the good fit of both the baseline and mediation models, including key fit indices such as CFI and TLI, which meet desirable levels. Although the RMSEA values suggest moderate absolute fit for both models, they fall within generally accepted ranges, indicating that the models can accurately describe and explain the underlying structural relationships of the studied phenomenon with high reliability (Wu, 2009). Comparing the results with stepwise regression, in the baseline model, teachers’ teaching strategies significantly predict students’ learning engagement positively (0.253). In the mediation model, both teachers’ teaching strategies and perceived teachers’ emotional engagement significantly predict students’ learning engagement positively in terms of direct effects (0.719 and 0.168, respectively).

**Table 3:** Mediation effect test of perceived teachers’ emotional engagement.

Variables	Direct effects model	Mediation model
Teachers’ teaching strategies	0.253^***^ (0.010)	0.719^***^ (0.117)
Perceived teachers’ emotional engagement		0.168^***^ (0.017)
RMSEA	0.66	0.61
GFI	0.965	0.926
AGFI	0.946	0.945
ECVI	0.234	0.288
NFI	0.957	0.960
RFI	0.945	0.950
IFI	0.958	0.961
TLI	0.946	0.951

Coefficients in the table are unstandardized coefficients, with standard errors in parentheses; model fit indices indicate adequate fit for both models; ^***^p < 0.001.

**Figure 2:**
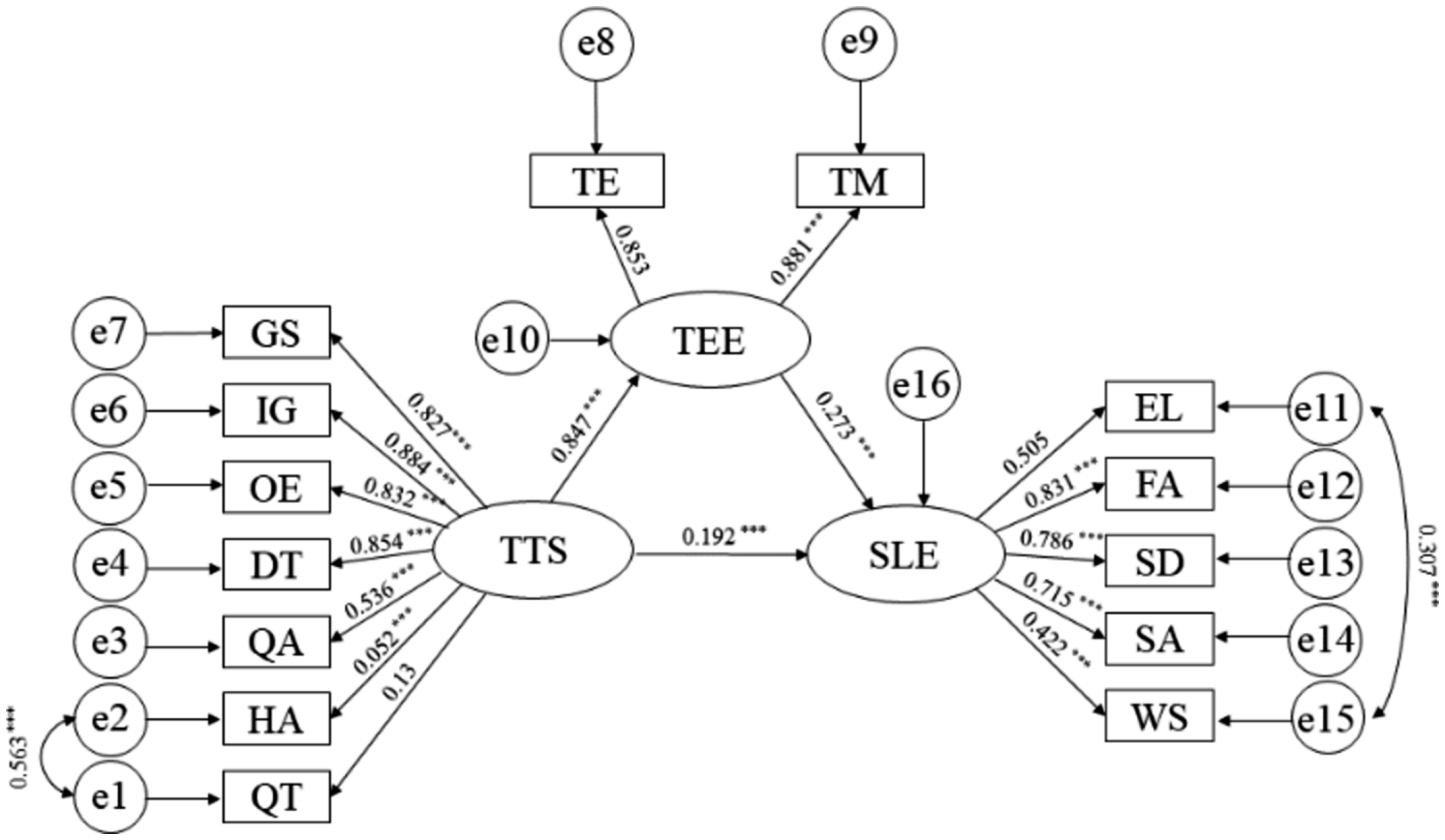
Structural equation model of student engagement. SLE, Students’ learning engagement; EL, Energy level; FA, Focus attention; SD, Self-discipline; SA, Self-awareness; WS, Willpower strength; TTS, Teachers’ teaching strategies; GS, Goal setting; IG, Inspiration guidance; OE, Oral evaluation; DT, Deep teaching; QA, Q & A; HA, Homework Assignment; QT, Quiz testing; TEE, Perceived teachers’ emotional engagement; TEE, Teacher Enthusiasm; TM, Teacher Motivation; TE, Teachers’ expectations; the coefficients marked in the figure are standardized coefficients; ***p < 0.001.

To provide a clearer representation of the influence of the mediating variables and their processes in this study, Figure 2 presents the path diagram of the mediation model, listing standardized path coefficients and their statistical significance.

As shown in Figure 2, teachers’ teaching strategies have a significant positive impact on perceived teachers’ emotional engagement (*p* < 0.001). Additionally, both teaching strategies and perceived teachers’ emotional engagement have significant positive impacts on students’ learning engagement (*p* < 0.001). In the influence pathway “Teachers’ teaching strategies → Perceived teachers’ emotional engagement → students’ learning engagement,” the direct effect of teaching strategies is 0.192 (*p* < 0.001), accounting for 45.37% of the total effect. The proportion of direct and indirect effects indicates the existence of an indirect (mediation) effect, which is 0.231, accounting for 54.63% of the total effect.

### Moderated mediation effect analysis

To test Hypothesis 3, Model 14 in Process v4.1 was utilized to examine the moderating effect of teacher expectations on the pathway “Teachers’ teaching strategies → Perceived teachers’ emotional engagement → Students’ learning engagement.” Gender, grade level, and family region were included as control variables in the moderated mediation model to eliminate the interference caused by demographic variables in this study. The results indicate that teachers’ expectations significantly moderate the pathway from “Perceived teachers’ emotional engagement” to “Student engagement” (see Table 4).

**Table 4:** Moderated mediation analysis.

Variables	Model 1 (DV=Students’ learning engagement)	Model 2 (DV=Perceived teachers’ emotional engagement)	Model 3 (DV=Students’ learning engagement)
Gender (female = 0)	−0.065^***^	−0.012	−0.065^***^
Grade	−0.125^***^	−0.157^***^	−0.077^***^
Region (rural = 0)	0.063^***^	0.124	0.056^***^
Teachers’ teaching strategies	0.487^***^	0.912^***^	0.222^***^
Perceived teachers’ emotional engagement			0.256^***^
Teachers’ expectations			0.054^***^
Perceived teachers’ emotional engagement × Teachers’ expectations			0.073^***^
*F*	457.386	1985.365	354.605
*R^2^*	0.169	0.470	0.212

^***^p < 0.001.

Using the non-parametric percentile Bootstrap method, teachers’ expectations were divided into three groups: low, medium, and high. The results (see Table 5) indicate that perceived teachers’ emotional engagement significantly increases with higher levels of teachers’ expectations. This suggests that teachers’ expectations play a facilitating role in the indirect effect of teaching strategies on students’ learning engagement.

**Table 5:** Bootstrap test results for mediated effects.

Teachers’ expectations	Effect	Bootstrap SE	Boot LLCI	Boot ULCI
M − 1SD	0.120	0.011	0.098	0.141
M	0.126	0.009	0.128	0.165
M + 1SD	0.173	0.012	0.149	0.198

**Figure 3:**
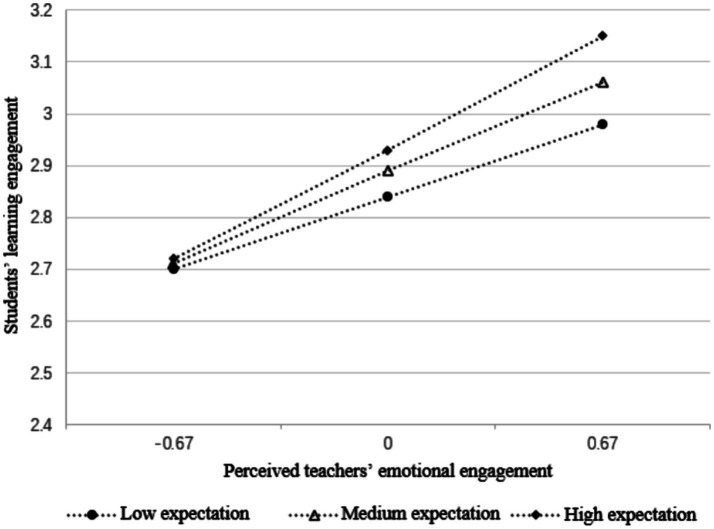
Moderation of teachers’ expectations on the relationship between perceived teacher emotional engagement and students’ learning engagement.

To visually present the moderation effect, a simple slope plot was created based on the analysis results from the Process macro. This plot illustrates the prediction of student engagement by perceived teachers’ emotional engagement for three groups of teachers’ expectations: low (Mean-SD), medium (Mean), and high (Mean + SD) (Figure 3).

Figure 3 demonstrates that when students perceive teachers’ lower expectations, the influence of perceived teachers’ emotional engagement on students’ learning engagement is minimal. Conversely, when students perceive higher expectations, the influence of perceived teachers’ emotional engagement on their learning engagement is significantly greater, indicating a substantial moderating effect. Hypothesis 3 is thus confirmed.

In summary, our analysis yields the following results: first, teachers’ teaching strategies have a significant positive predictive effect on students’ learning engagement; second, students’ perceived teachers’ emotional engagement mediates the effect of teachers’ teaching strategies on their students’ learning engagement; third, teachers’ expectations moderate the latter part of the pathway “teaching strategies → perceived teachers’ emotional engagement → students’ learning engagement,” specifically the relationship between perceived teachers’ emotional engagement and students’ learning engagement.

## Discussion

This study identifies a significant positive correlation between teachers’ teaching strategies and students’ learning engagement, consistent with existing research (Cents-Boonstra et al., 2021; Mitchell, 2014). Cents-Boonstra et al. evaluated the relationship between teaching strategies and student engagement, finding a positive correlation where teaching strategies enhance students’ classroom participation in supportive and guided classroom environments. Mitchell argued that teaching strategies such as teacher-student interaction, peer interaction, and feedback can increase students’ sense of involvement in learning. These consistent findings affirm the impact of teachers’ teaching strategies on students’ learning engagement. Moreover, they can also be explained through social cognitive theory. According to social cognitive theory (Schunk and Usher, 2012), when teachers employ diverse teaching strategies based on students’ actual needs and interests, it stimulates students’ interest in learning and encourages them to engage more actively in their studies. Hypothesis 1 proposed in this study (that diverse teaching strategies enhance students’ learning engagement) receives further validation and support.

This study found that perceived teachers’ emotional engagement partially mediates the relationship between teachers’ teaching strategies and students’ learning engagement. On one hand, teaching strategies can directly predict student engagement; on the other hand, teaching strategies can indirectly influence students’ engagement through perceived teachers’ emotional engagement. In this study, a one standard deviation change in teaching strategies results in a 0.423 standard deviation change in students’ learning engagement, with 0.192 attributable to the direct effect of teaching strategies on students’ learning engagement and 0.231 attributable to the indirect effect mediated by perceived teachers’ emotional engagement. When teachers employ diverse teaching strategies, students perceive positive emotional engagement from the teachers, which fosters positive emotions, forms a positive learning attitude, and promotes students’ learning engagement. This result is consistent with other research findings (Frenzel et al., 2009; Dewaele and Li, 2021; Kuo et al., 2024).

This study contributed to a moderated mediation model involving teaching strategies, perceived teachers’ emotional engagement, students’ learning engagement, and teachers’ expectations, thus validating Hypothesis 3. Teachers’ expectations moderated the latter part of the mediation process in the pathway “teaching strategies → perceived teachers’ emotional engagement → students’ learning engagement.” In this study, teachers’ expectations were measured by the extent to which students perceived that their teachers cared about their learning. According to the Pygmalion effect, students who believe that their teachers have higher expectations of them generally exhibit better learning attitudes and thus better academic performance (Rosenthal and Jacobson, 1966; Kong, 2011). Students with better performance may gain more support from teachers and thus establish better relationships (Hughes and Kwok, 2007). This positive relationship between teachers and students helps students to further transform the positive emotions they feel from teachers’ teaching behaviors into their own learning motivation, resulting in higher engagement (Martin and Collie, 2019). On the contrary, if students believe that they are not expected, their learning engagement and academic performance will be inhibited, and the quality of teacher-student relationship will be affected, which will again affect students’ learning engagement and academic performance (Fowler et al., 2008). This partly explains why students’ perception of teachers’ emotional engagement can significantly predict students’ learning engagement.

This innovation of this research lies in the establishment of a moderated mediation model, which provides an interesting understanding of the underlying mechanisms by which teaching strategies influence students’ learning engagement, rather than merely affirming the existence of this influence. According to the model, effective teaching strategies exert both a direct impact on students’ learning engagement and an indirect impact mediated by students’ perceptions of teachers’ emotional engagement. Crucially, the extent to which students perceive teachers’ expectations moderates this indirect effect. Students who perceive higher teachers’ expectations are more adept at translating perceived teachers’ emotional engagement into learning engagement. In contrast, students who perceive lower expectations are less effective in this translation process. This finding suggests that the level of teachers’ expectations influences the efficacy with which students convert emotional perceptions into engagement behaviors. Thus, student learning engagement is not only related to internal emotional and affective factors but is also shaped by external environmental factors (Li and Xue, 2023).

## Implications and conclusion

This study yields several key insights. First, given the strong relationship between teachers’ teaching strategies and students’ learning engagement, teachers should employ captivating teaching strategies to effectively capture students’ attention, stimulate their curiosity, and enhance both learning engagement and satisfaction (Garrison and Vaughan, 2008). It is also essential for teaching strategies to be diverse and multifaceted to cater to the varied needs of different students.

Second, students not only acquire knowledge during the learning process but also form emotional responses and learning attitudes by observing and perceiving their teachers’ emotional states (Pellitteri and Smith, 2007). While teachers can convey positive emotions through rich teaching strategies in offline settings (Sagayadevan and Jeyaraj, 2012), this emotional transmission can also transcend spatial boundaries in teaching process, shaping a positive classroom atmosphere and enhancing students’ emotional experiences (Kim and Hodges, 2012). The effective transmission of teachers’ enthusiasm and passion embedded in their teaching strategies significantly promotes student’s class engagement (Dewaele and Li, 2021). Therefore, utilizing technologies such as augmented reality to achieve visualized teaching can enhance emotional transmission, create a positive emotional environment (Garzón and Acevedo, 2019; Zeinstra et al., 2023), optimize the pathways for emotional conveyance, and facilitate the mediation role of perceived teachers’ emotional engagement. This encourages students to convert perceived positive emotions into positive learning attitudes and diligent learning behaviors.

Third, teachers should place greater emphasis on the impact of their attention and expectations on students. On one hand, teachers should clearly express high expectations for their students, as such positive expectations can serve as a crucial motivator, encouraging students to set higher personal learning goals and thus increasing their proactivity and engagement in learning (ACT Government, 2019; Jussim and Harber, 2005). On the other hand, teachers need to provide personalized feedback and encouragement, offering appropriate recognition and adjusting expectations based on each student’s abilities and progress (Lehtinen et al., 2023). Ensuring that every student feels valued and seen can further enhance their sense of involvement and achievement in learning (Bernard et al., 2019). In summary, teaching is not merely a process of knowledge transmission but also an emotional and affective endeavor. The effective conveyance of positive emotions and the establishment of emotional connections can significantly foster student engagement in learning activities.

This study identifies significant positive correlations between teachers’ diverse teaching strategies in online teaching environments and students’ learning engagement. It further reveals that perceived teachers’ emotional engagement mediates the relationship between teaching strategies and students’ engagement, while teachers’ expectations moderate the link between perceived teachers’ emotional engagement and students’ engagement. This enriches the research on how teachers’ teaching practices impact students’ learning, offering new perspectives and insights for understanding and optimizing teaching practices to enhance student learning outcomes. Despite providing new empirical evidence for understanding the relationship between teachers’ teaching strategies and students’ learning engagement in online teaching environments, this study has several limitations. Firstly, the data were collected exclusively from primary and secondary school students in one province, which may restrict the generalizability of the findings. Future research should include a broader range of participants from various countries and administrative regions to verify the universality of the current results (Babbie, 2020). Secondly, the study employed a cross-sectional design, making it difficult to uncover the dynamic relationships between teaching strategies and learning engagement. Future studies could utilize longitudinal or mixed-method research designs and incorporate multimodal data collection methods to explore the long-term dynamic relationships and causal mechanisms between teaching strategies and learning engagement.

## Limitation

In this study, a micro-scale containing two items was used to measure students’ perception of teachers’ emotional engagement. Although it has good reliability and does not affect the core issues to be discussed in this study, the content validity of conceptual measurement has certain limitations due to the small number of items in the scale.

## Data Availability

The original contributions presented in the study are included in the article/supplementary material, further inquiries can be directed to the corresponding author.
